# Supplementary material for: An Individualized Low-Pneumoperitoneum-Pressure Strategy May Prevent a Reduction in Liver Perfusion during Colorectal Laparoscopic Surgery
Source: Biomedicines. 2023 Mar 14;11(3):891. doi: 10.3390/biomedicines11030891 (PMC10045598; doi:10.3390/biomedicines11030891)
Supplement: Supplementary file 1 [file biomedicines-11-00891-s001.zip › biomedicines-2189564-supplementary.docx]

**Supplementary Material**

**Table S1.** Multivariable beta regression model estimation with ICG-PDR as dependent variable.

| **Variables** | **Estimate** | **Std. Error** | **Odds Ratio**  **[95%CI]** | ***p-*value** |
| --- | --- | --- | --- | --- |
| **Intercept** | −0.93 | 0.538 | 0.39 [0.14 to 1.13] | 0.082 |
| **IPP group** | 0.35 | 0.13 | 1.42 [1.10 to 1.82] | 0.006 |
| **ASA (ref. ASA I)** |  | | | |
| ASA II | −0.54 | 0.19 | 0.59 [0.40 to 0.85] | 0.005 |
| ASA III | −0.75 | 0.23 | 0.47 [0.30 to 0.74] | 0.001 |
| **Age (years)** | −0.004 | 0.01 | 0.99 [0.99 o 1.01.] | 0.346 |
| **Female** (yes) | −0.084 | 0.19 | 0.92 [0.63 to 1.33] | 0.658 |
| **BMI (kg ∙ m^−2^)** | 0.001 | 0.02 | 1.01 [0.97 to 1.03] | 0.960 |
| **Rectal surgery** (yes) | −0.108 | 0.13 | 0.90 [0.70 to 1.15] | 0.399 |
| **phi** | 97.84 |  | | |
| **Pseudo R^2^** | 0.56 |  | | |

Abbreviatons: IPP, Individualized Pneumoperitoneum Pressure; ASA, American Society of anesthesiology; BMI, Body Mass Index

**Table S2.** Multivariable beta regression model estimation with ICG-R_15_ as dependent variable.

| **Variables** | **Estimate** | **Std. Error** | **Odds Ratio**  **[95%CI]** | ***p-***  **value** |
| --- | --- | --- | --- | --- |
| **Intercept** | −4.2 | 1.13 | 0.01 [0.01 to 0.13] | >0.001 |
| **IPP group** | −0.78 | 0.24 | 0.46 [0.28 to 0.73] | 0.001 |
| **ASA (ref. ASA I)** |  | | | |
| ASA II | 1.17 | 0.55 | 3.23 [1.10 to 9.51] | 0.033 |
| ASA III | 1.59 | 0.61 | 4.92 [1.50 to 16.07] | 0.008 |
| **Age (years)** | −0.01 | 0.01 | 0.99 [0.98 o 1.02] | 0.939 |
| **Female** (yes) | 0.10 | 0.31 | 1.11 [0.60 to 2.04] | 0.730 |
| **BMI (kg ∙ m^−2^)** | 0.04 | 0.03 | 1.04 [0.98 to 1.11] | 0.187 |
| **Rectal surgery** (yes) | 0.14 | 0.23 | 1.15 [0.72 to 1.82] | 0.542 |
| **phi** | 38.84 |  | | |
| **Pseudo R^2^** | 0.53 |  | | |

Abbreviatons: IPP, Individualized Pneumoperitoneum Pressure; ASA, American Society of
anethesiology; BMI, Body Mass Index.

**Complete list of the** **IPPColLapSe II investigators:**

Oscar Díaz-Cambronero ([oscardiazcambronero@gmail.com)](mailto:oscardiazcambronero@gmail.com))

Blas Flor-Lorente ([blasflor@hotmail.com)](mailto:blasflor@hotmail.com))

Guido Mazzinari ([gmazzinari@gmail.com)](mailto:gmazzinari@gmail.com))

María Vila-Montañés ([mvilamontanes@yahoo.es)](mailto:mvilamontanes@yahoo.es))

Nuria García-Gregorio ([nuriagcia6@gmail.com)](mailto:nuriagcia6@gmail.com))

Maria Jose Alberola-Estellés ([majoni2000@yahoo.es)](mailto:majoni2000@yahoo.es))

Begoña Ayas-Montero ([bego_ayas@hotmail.com)](mailto:bego_ayas@hotmail.com))

Salome Matoses-Jaén ([smatosesj@gmail.com)](mailto:smatosesj@gmail.com))

Sandra Verdeguer ([sandraverdeguer@hotmail.com)](mailto:sandraverdeguer@hotmail.com))

Anabel Marqués Marí ( [amarquesmari@gmail.com)](mailto:amarquesmari@gmail.com))

Jose Miguel Alonso Íñigo (jmalonso@me.com)

Josep Balaguer Domenech ([jbalaguer.doc@gmail.com)](mailto:jbalaguer.doc@gmail.com))

Marisol Echeverri Velez (maechevelez@hotmail.com)

David Cuesta-Frau (dcuesta@disca.upv.es)

Maria Pilar Argente-Navarro ([argente_marnav@gva.es)](mailto:argente_marnav@gva.es))

Salvador Pous ([salvadorpous@hotmail.com)](mailto:salvadorpous@hotmail.com))

Cristina Ballester ([cris7balle@yahoo.es)](mailto:cris7balle@yahoo.es))

Matteo Frasson ([dr.frasson.matteo@gmail.com)](mailto:dr.frasson.matteo@gmail.com))

Alvaro García-Granero ([alvarogggt@hotmail.com)](mailto:alvarogggt@hotmail.com))

Carlos Cerdán-Santacruz ([carloscerdansantacruz@hotmail.com)](mailto:carloscerdansantacruz@hotmail.com))

Eduardo García-Granero ([eggranero@telefonica.net)](mailto:eggranero@telefonica.net))

Luis Sánchez-Guillén ([drsanchezguillen@gmail.com)](mailto:drsanchezguillen@gmail.com))

Daniel Robles-Hernández ([drobher@gmail.com)](mailto:drobher@gmail.com))

David Boquera-Albert ([david.boquera@gmail.com](mailto:david.boquera@gmail.com))

David Casado-Rodrigo ([dcasador@hotmail.com](mailto:dcasador@hotmail.com))

Rebeca Cosa-Rodríguez ([rebecacosa@hotmail.com](mailto:rebecacosa@hotmail.com))

Luis Enrique Olmedilla-Arnal ([lolmedilla@gmail.com)](mailto:lolmedilla@gmail.com))

Marcos Rodríguez-Martín ([marcosrodmar@hotmail.com)](mailto:marcosrodmar@hotmail.com))

Jaime Zorrilla-Ortúzar (jzorrillaortuzar@gmail.com)

José María Pérez-Peña ([jppena@salud.madrid.org)](mailto:jppena@salud.madrid.org))

Ángel Martín-de-Pablos (angelmartindepablos@gmail.com)

Javier Valdés-Hernández ([cirugia@drjaviervaldes.com](mailto:cirugia@drjaviervaldes.com))

Juan Carlos Gómez-Rosado ([juanc.gomez.rosado.sspa@juntadeandalucia.es](mailto:juanc.gomez.rosado.sspa@juntadeandalucia.es))

Pino Heredia-Pérez ([pinohp@hotmail.com](mailto:pinohp@hotmail.com))

Juan Cintas-Catena ([juancintascatena@gmail.com](mailto:juancintascatena@gmail.com))

Fernando Flor-Parra ([fernandoflorp@gmail.com](mailto:fernandoflorp@gmail.cm))

Marcus J. Schultz ([marcus.j.schultz@gmail.com)](mailto:marcus.j.schultz@gmail.com))

Carlos Luis Errando Oyonarte ([errando013@gmail.com)](mailto:errando013@gmail.com))
